# NeoSLAM: Long-Term SLAM Using Computational Models of the Brain

**DOI:** 10.3390/s24041143

**Published:** 2024-02-09

**Authors:** Carlos Alexandre Pontes Pizzino, Ramon Romankevicius Costa, Daniel Mitchell, Patrícia Amâncio Vargas

**Affiliations:** 1 PEE/COPPE—Department of Electrical Engineering, Federal University of Rio de Janeiro, Cidade Universitária, Centro de Tecnologia, Bloco H, Rio de Janeiro 21941-972, RJ, Brazil; ramon@coep.ufrj.br; 2 Edinburgh Centre for Robotics, Heriot-Watt University, Edinburgh EH14 4AS, UK; d.mitchell.5@research.gla.ac.uk (D.M.); p.a.vargas@hw.ac.uk (P.A.V.)

**Keywords:** long-term visual SLAM, biologically inspired robots, neurorobotics, sparse distributed representation, hierarchical temporal memory

## Abstract

Simultaneous Localization and Mapping (SLAM) is a fundamental problem in the field of robotics, enabling autonomous robots to navigate and create maps of unknown environments. Nevertheless, the SLAM methods that use cameras face problems in maintaining accurate localization over extended periods across various challenging conditions and scenarios. Following advances in neuroscience, we propose NeoSLAM, a novel long-term visual SLAM, which uses computational models of the brain to deal with this problem. Inspired by the human neocortex, NeoSLAM is based on a hierarchical temporal memory model that has the potential to identify temporal sequences of spatial patterns using sparse distributed representations. Being known to have a high representational capacity and high tolerance to noise, sparse distributed representations have several properties, enabling the development of a novel neuroscience-based loop-closure detector that allows for real-time performance, especially in resource-constrained robotic systems. The proposed method has been thoroughly evaluated in terms of environmental complexity by using a wheeled robot deployed in the field and demonstrated that the accuracy of loop-closure detection was improved compared with the traditional RatSLAM system.

## 1. Introduction

A Simultaneous Localization and Mapping (SLAM) method allows a robot to continuously create a map of the environment and at the same time estimate its location based on this map [1]. Since robots can be applied sector-wide, it is important that robots can safely and accurately localize themselves with the environment to ensure efficient operation in challenging sectors, such as the Offshore Renewable Energy (ORE) sector [2,3,4], Nuclear sector [5,6], and Medical sector [7].

One of the most important aspects of a SLAM system is place recognition or loop-closure detection (LCD). This aims to reduce the robot pose uncertainty due to the effect of the errors introduced by the odometry. Moreover, the incorrect data associations in the LCD can result in a critical failure for SLAM algorithms [8].

**Figure 1:**
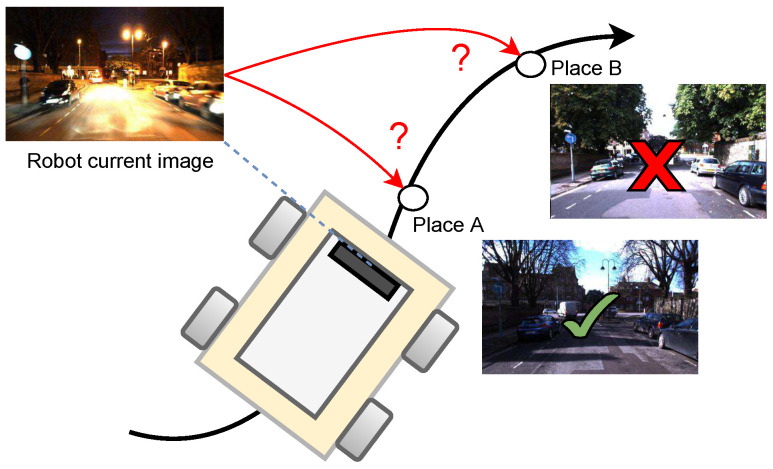
In a SLAM system, the goal of VPR might be to find the matching image between the current image and database images for loop-closure detection. The figure serves as a compelling illustration of the need to address the problem.

As the usage of vision sensors has increased rapidly, the visual place recognition (VPR) task has been widely studied in recent years. Cameras are cheaper than laser scanners and provide increasing amounts of information. By definition, a VPR system must be able to recognize a previously visited place via visual information [9]. In other words, an autonomous robot that operates in an environment should be able to recognize different places when it revisits them after some time as in long-term robot operation (Figure 1).

The implementation of feature-extraction techniques is a vital phase in VPR systems as it involves recognizing and describing distinct patterns or indicators in an image and then using them for matching and recognition. Most of the state-of-the-art methods can be broadly categorized into two types based on the nature of image-wise descriptors: local and global descriptors. Local image descriptors focus on extracting distinctive features from key points in the image (e.g., Scale-Invariant Feature Transform (SIFT) [10], Speeded-Up Robust Features (SURF) [11], Oriented FAST and Rotated BRIEF (ORB) [12], and Binary Robust Invariant Scalable Keypoints (BRISK) [13]. On the other hand, global image descriptors aim to capture a holistic representation of the entire scene (e.g., Bag-of-Visual-Words (BoVW) [14], Convolutional Neural Network (CNN) [15], Fisher Vectors [16], and Vector of Locally Aggregated Descriptors (VLAD) [17].

**Figure 2:**
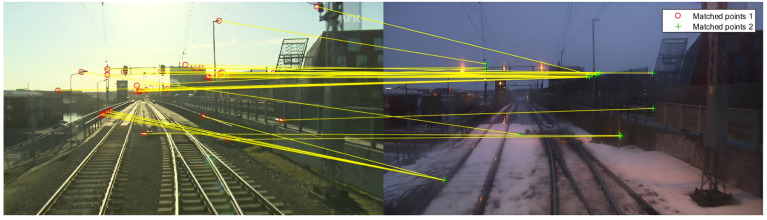
Speeded-Up Robust Features (SURF) [11] applied to Nordland dataset [19] (licensed under Creative Commons) for daytime versus evening. While effective in certain scenarios, SURF may struggle when confronted with significant changes in illumination, as it relies on local features that are not inherently invariant to lighting variations.

Although many studies have been conducted recently, there are still open questions regarding the long-term operation of robots [18], as can be seen in Figure 2.

Long-term SLAM models need to be robust to changes in the robot’s environment caused by different conditions (day–night cycles, changing weather, and so on), occlusion, and viewpoints [20] and deal with the stability–plasticity dilemma, which is a concept in neuroscience and machine learning. This dilemma refers to the challenge of finding the right balance between stability and plasticity in learning systems. Whereas stability refers to the ability of a system to maintain existing knowledge, plasticity refers to adapting and learning from new experiences [18].

Inspired by neuroscience discoveries, various brain-inspired methodologies have been proposed and demonstrated that the analysis of animal behaviors and the biological process of navigation and mapping can produce interesting insights for robotic applications in the world under extremely different environmental conditions. In [21,22,23], Milforld et al. proposed the RatSLAM system, which includes a bioinspired SLAM system based on computational models of the rodent hippocampus. The system is based on a Continuous Attractor Neural Network (CANN) and can construct a cognitive map through low-resolution monocular image data and odometry information. In [24], Silveira et al. proposed the DolphinSLAM inspired by the RatSLAM. This SLAM system is a bioinspired algorithm for underwater robots using probabilistic local features based on the data-association method of the Fast-Appearance-Based Mapping technique (FAB-MAP). In [25], Yuan et al. proposed an entorhinal–hippocampal model with high biological fidelity, which is able to build cognitive maps simultaneously by integrating activities of place cells and grid cells with visual inputs. In [26], Lu et al. proposed a full visual SLAM system combining biologically inspired visual odometry and RatSLAM. In [27], Kazmi et al. introduced Gist+RatSLAM, a framework that integrates the Gist descriptor into the RatSLAM pipeline. In [28], Zhou et al. adopted the RatSLAM algorithm’s vision processing based on the Oriented FAST and Rotated BRIEF (ORB) feature-extraction approach. In [29], Zeng et al. proposed a model based on conjunctive head-direction-by-velocity and conjunctive grid-by-velocity cells to integrate movement and sensory information. In [30], Yu et al. proposed a neuroinspired SLAM system, namely NeuroSLAM, which integrates 3D grid cell models and multilayered head-direction cell models based on RatSLAM. In [31], the authors presented an unsupervised learning framework for multisensor representation that yields low-dimensional latent state descriptors that can be used for RatSLAM. In [32], Kasebi et al. used the Scale-Invariant Feature Transform (SIFT) algorithm to improve the visual matching of the RatSLAM system.

Although these works use biologically inspired SLAM methods in order to perform inference based on the data produced by the front end, they do not bring benefits from neuroscience-based methods to visual place recognition and loop-closure tasks. For instance, the visual template feature is organized as a one-dimensional vector whose values only depend on pixel intensity in the RatSLAM algorithm, and this feature is susceptible to changes in illumination intensity.

In recent years, there has been an increasing amount of literature on the integration of biological and neuroscientific principles into the development of visual place recognition algorithms. In [33], Fen et al. investigated multiscale grid cells observed in the mammalian brain for performing visual place recognition. In [34], Neubert et al. explored the relationships between place recognition and HTM theory and presented the Simplified Higher Order Sequence Memory (SHOSM) algorithm. This neurally inspired model is a simplified version of the HTM framework for place recognition. A significant analysis and discussion on the subject were presented by the authors. This theoretical research was then applied to real-world data in combination with CNN-based image descriptors in [35]. Pizzino et al. demonstrated that the usage of the framework originally proposed by [36] can extend the run time during long-term operations when compared to [35]. The outcomes indicate that the suggested architecture is capable of encoding an internal representation of the world by employing a fixed number of cells, thereby enhancing system scalability. In [37], J. Li et al. proposed a loop-closure detection by using a neural hashing algorithm inspired by the fly olfactory circuit, which is applied to a hippocampal–entorhinal-based SLAM system to build cognitive maps with improved robustness and effectiveness.

In this paper, we present a novel visual SLAM method that integrates computational models inspired by the neocortex and hippocampal–entorhinal regions. This study set out to assess the feasibility of implementation in robots that operate in dynamic environments characterized by continuously changing appearances. The usage of Spatial Distributed Representations (SDRs), which are known to be representations in the brain, enables real-time performance and allows for efficient memory storage. A CNN-based encoder is used to promote the robustness of matching for correspondences in the images. Based on the neocortex model, the network gradually and continually adapts with each new input. The system was validated and verified via the deployment of the SLAM system onboard real-world ground-based robots. The environments where the evaluation of the system took place consisted of a university campus with outdoor and indoor areas and a farm yard that included several animals within a barn.

A summary of the contributions of this work includes the following:

- We present NeoSLAM, a novel long-term visual SLAM that integrates computational models of the neocortex and hippocampal–entorhinal system in order to enhance the efficiency and robustness to changes in the robot’s environment caused by different conditions.
- A new loop-closure detector based on spatial-view cells is presented. The method uses binary sparse distributed representations, offering a compact and powerful way to encode complex patterns in data. Unlike traditional dense representations, which require substantial computational resources, our method significantly reduces the computational load, making it particularly suitable for real-time applications.
- We provide a thorough experimental evaluation that involves deploying the system in practical scenarios, enabling a thorough examination of its performance and capabilities within the Robot Operating System (ROS) framework.

This paper proceeds as follows: Section 2 presents the architecture and the detailed model of our NeoSLAM method. Section 3 describes the design of the experiments for investigating and evaluating the performance of NeoSLAM. Section 4 and Section 5 provide analyses of the results and suggestions for future work, respectively.

## 2. NeoSLAM

Over recent years, the interaction between the neocortex and the hippocampus has been investigated and serves as inspiration for our method. Both brain parts are critical regions in the primate brain and work together to cope with various cognitive functions, particularly those related to learning and spatial memory [38].

The neocortex is the outer layer of the brain and is responsible for higher-order cognitive processes, such as perception, language, and conscious thought. It is involved in processing sensory information and integrating it with other information to form complex representations of the world. On the other hand, the hippocampus is a seahorse-shaped structure located deep within the brain underneath the neocortex and is primarily associated with memory formation and spatial navigation. In primates, the hippocampus receives major input via the entorhinal cortex. These inputs come from the ends of many processing streams of the cerebral association cortex, including the visual cortical areas [39].

Similar to rodents, primate place cells, also referred to as spatial-view cells, exhibit selective firing patterns associated with specific locations in the environment. However, primate spatial-view cells often have larger receptive fields, meaning they are active in broader areas of space compared to the more precise place fields of rodents. The firing patterns of primate spatial-view cells are not limited to specific locations alone. They can also show specificity to other spatial features, such as landmarks or boundaries. For example, some spatial-view cells may fire preferentially when the animal is near a particular visual landmark while others may be sensitive to the boundaries of the environment [40].

**Figure 3:**
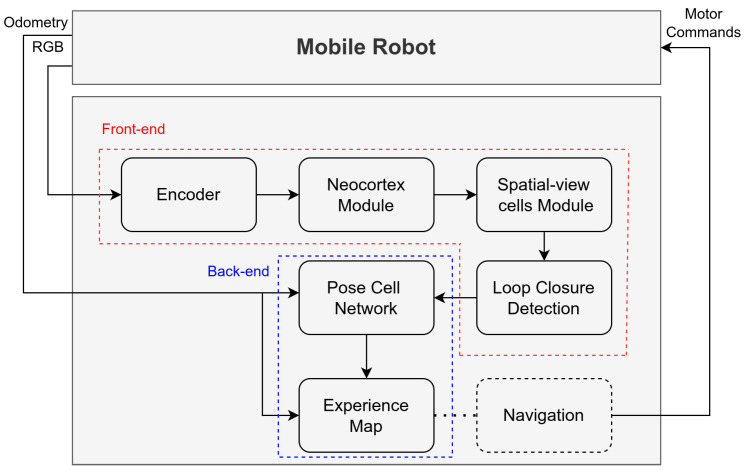
An overview of the major modules of the NeoSLAM system. Most SLAM approaches are commonly divided into two main components: the front-end and the back-end blocks. The former is responsible for real-time sensor data processing, encompassing tasks such as feature extraction and establishing correspondences between consecutive frames. This component plays a crucial role in tracking the system’s pose and understanding the environment. On the other hand, the latter focuses on optimization and map refinement. It integrates the accumulated sensor measurements over time, corrects for errors, and optimizes the estimated trajectory and map. In our system, both components draw inspiration from neuroscience models.

Based on theories and models of neuroscience, NeoSLAM allows the robot to perform SLAM in real time by recognizing places it has previously visited under variations in appearance and illumination. The system consists of five major modules, namely the encoder, neocortex, spatial-view cells and LCD, pose cells, and experience map, as can be seen in Figure 3. First, we present the neocortex model, and after that, the other parts.

### 2.1. Neocortex Model

In this work, we use one framework that models a number of structural and algorithmic properties of the neocortex, namely hierarchical temporal memory (HTM) proposed by Hawkins in [41]. In this framework, the brain processes information by using sparse distributed representations (SDRs).

#### 2.1.1. Sparse Distributed Representations

The neocortex does not directly receive photons or vibrations from the external environment. Before entering the brain for processing, sensory signals need to be converted into a shared representation space. This widely employed representation space in the mammalian brain is referred to as sparse distributed representations (SDRs).

Sparse neuronal activity refers to the phenomenon observed in mammalian brains where only a small fraction of neurons are active at any given time, while the majority remain silent or exhibit low firing rates. This pattern of sparsity is a fundamental characteristic of neural processing and has been extensively studied to understand how information is represented and processed in the brain.

One key advantage of sparse neuronal activity is its efficiency in representing information. By activating only a subset of neurons, the brain can achieve high-capacity representations while minimizing energy consumption and neural resources. This sparsity also enables the selective and precise encoding of relevant features or stimuli, allowing the brain to efficiently extract and process salient information from the environment.

Given a population of *n* neurons or binary units, their instantaneous activity is represented as an SDR, i.e., an *n*-dimensional vector of binary components:

(1) $$x=[b_{0},\cdot \cdot \cdot ,b_{n-1}],$$

where $x\in S:{\left\{1,0\right\}}^{n}$ and a small percentage of the components are one. The number of components in *x* that are one is defined as $\omega_{x}={∥x∥}_{1}$.

The similarity between two SDR vectors is determined by an overlap score, i.e., the number of bits that are one in both vectors. Let Φ:S×S→N be a function defined by

(2) Φ=x·y,

where $x,y\in S^{n}$. The overlap score is simply the number of bits that are one in the same locations.

A match between two SDR vectors occurs if their overlap exceeds the threshold β, i.e.,

(3) Ψ=Φ(x,y)≥β.

Typically β is set such that $\beta \leq \omega_{x}$ and $\beta \leq \omega_{y}$.

#### 2.1.2. Notation

**Figure 4:**
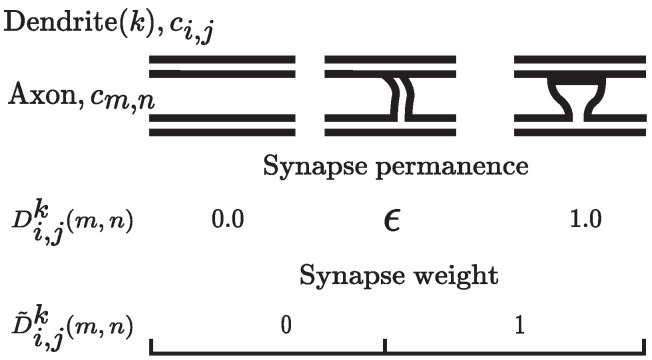
A permanence value is assigned to each potential synapse and represents the growth of the synapse. It is a key parameter that regulates the strength of connections between neurons and allows the model to adapt and encode relevant patterns in the input space over time. Synapses are subject to both potentiation and depression. If a synapse is active and contributes to the cell’s activation, its permanence value may be increased. Conversely, its permanence value may be decreased.

Let *n* represent the number of minicolumns in the layer, *m* the number of cells per column, and nm the total number of cells in the layer. A single-level network structure consists of one region that is arranged in minicolumns $C_{j}$, (j=1,2,3,⋯,n), that have multiple cells $c_{i,j}$, (i=1,2,3,⋯,m). In this work, the following terminology for describing the algorithms is used:

- Cell state: each cell $c_{i,j}$ can be in an active state, in a predictive (depolarized) state, or in a nonactive state.
- Active state: Matrix of active cells, $A^{t}=\left\{a_{i,j}^{t}\right\}$, where $a_{i,j}^{t}$ is the active state of the *i*’th cell in the *j*’th column at any time step *t*. A value of 1 indicates an active state and a value of 0 indicates an inactive state.
- Predictive state: Matrix of predictive cells, $\Pi^{t}=\left\{\pi_{i,j}^{t}\right\}$, where $\pi_{i,j}$ is the predictive state of the *i*’th cell in the *j*’th column at any time step *t*. A value of 1 indicates a predicted state and a value of 0 indicates an unpredicted state.
- Winner cells: Matrix of winner cells, $W^{t}=\left\{w_{i,j}^{t}\right\}$, where $w_{i,j}^{t}$ is the winner *i*’th cell in the *j*’th column at any time step *t*. A winner cell is an active cell that was predicted or selected from the bursting minicolumn.
- Minicolumn state: Each minicolumn $C_{j}$ has a binary state variable $A_{j}^{t}$, where a value of 1 indicates an active state and a value of 0 indicates an inactive state.
- Dendrite segments: Each cell $c_{i,j}$ has one proximal dendrite segment and one or more distal dendrite segments. The proximal dendrite segment is a single shared dendrite segment per each minicolumn $C_{j}$ of cells and receives feed-forward connections $F_{j}$ from dimensions of the input *I*. The distal dendrite segments receive lateral input from nearby cells through the synapses on each segment.
- Synapse: Connection between an axon of one neuron and a dendrite of the other. The dendrite segments contain a number of potential synapses that have an associated permanence value. The permanence value of a synapse is a scalar value ranging from 0.0 to 1.0. If the permanence value of the potential synapse is greater than a threshold ϵ, it becomes a functional synapse, as can be seen in Figure 4. In HTM theory, synapses have binary weights.

Let $D_{i,j}^{k}=\left\{d_{i,j}^{k}\right\}$ be a set of distal segments that represents the *k*’th segment of the cell $c_{i,j}$ used to store the synaptic permanence value. The matrix of dendrite branch connectivity, ${\tilde{D}}_{i,j}^{k}=\left\{{\tilde{d}}_{i,j}^{k}\right\}$, is defined as

(4) $${\tilde{d}}_{i,j}^{k}=\left\{\begin{array}{cc} 1, & \mathrm{if}\;d_{i,j}^{k}\geq \epsilon \\ 0, & \mathrm{otherwise} \end{array}\right.$$

The matrix of positive terms of dendrite branch synapses, ${\dot{D}}_{i,j}^{k}=\left\{{\dot{d}}_{i,j}^{k}\right\}$, is defined by

(5) $${\dot{d}}_{i,j}^{k}=\left\{\begin{array}{cc} 1, & \mathrm{if}\;d_{i,j}^{k}\geq 0\\ 0, & \mathrm{otherwise} \end{array}\right.$$

- Learning: the process of learning involves incrementing or decrementing the permanence values ϵ of potential synapses on a dendrite segment.

#### 2.1.3. Hierarchical Temporal Memory

Hierarchical temporal memory (HTM) [42] is a neural network model designed to simulate the structure and function of the human neocortex. It is particularly designed to perform pattern recognition and anomaly detection and exhibits three key properties: sequence learning, continual learning, and sparse distributed representations.

The HTM algorithm is based on the idea that the neocortex learns and recognizes patterns through a hierarchical structure of neurons that process information in a sequence of temporal patterns. This structure allows the neocortex to detect and learn complex patterns over time and make predictions based on past experiences.

The HTM algorithm mimics this structure by creating a hierarchical structure of nodes, where each node represents a pattern or sequence of patterns. The nodes are connected to each other in a way that allows them to learn and recognize patterns over time. The algorithm also includes mechanisms for learning and adapting to new patterns in the input data.

One of the key advantages of the HTM algorithm is its ability to handle noisy and incomplete data. The hierarchical structure allows the algorithm to detect and learn from patterns even when the input data are incomplete or contain errors. The algorithm can also adapt to changes in the input data and learn new patterns without requiring a lot of retraining.

The HTM cells are stacked into columns, where groupings form an HTM region. The region replicates the structure and operations of the cortical column in the neocortex. Cells within a region gather information from three distinct sources: feedforward, contextual, and feedback information is conveyed through three separate connections.

The cortical columns within the neocortex are commonly denoted as minicolumns or columns. The fundamental unit in HTM is the cell. Minicolumns comprise multiple cells, and the network space of HTM is constituted by a substantial number of minicolumns.

The cells can be in inactive, active, or predicted states where segments (links to cells) comprise a collection of synapses. The synapses allow for information to be shared with the cells where, in HTM, this information is displayed as binary values. The information sent to the cell will determine the state. Processing is conducted by a Spatial Pooler algorithm for feedforward input, and temporal memory is used to provide contextual input. It is suggested that feedback is an optional component and is not currently addressed as per HTM theory [43].

**Figure 5:**
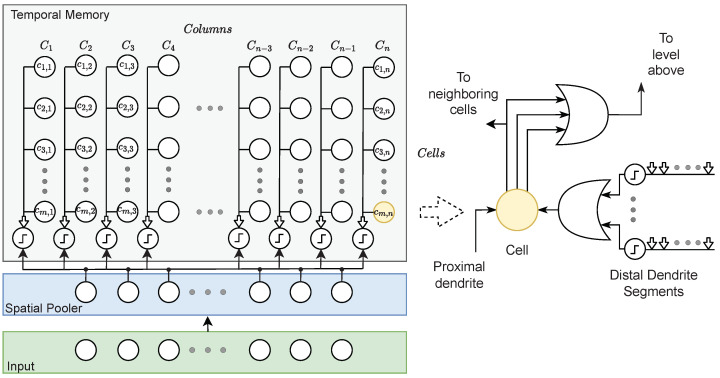
The cells within a column possess a collective proximal dendrite corresponding to the input space through a series of synapses illustrated as arrows. A cell is portrayed along with its distal dendrites on the right side where each dendrite segment establishes multiple synaptic connections with other cells.

Figure 5 shows the HTM model and includes two modules: Spatial Pooler (SP) and temporal memory (TM).

The main role of the Spatial Pooler (SP) in HTM theory is finding spatial patterns in the input data and transforming them into SDRs in a continuous online fashion. It may be decomposed into three stages: overlap calculation, inhibition, and learning [44]. In the case of visual localization, SP acts as a feature detector and extracts distinctive properties of a place that can be used to recognize this place [34].

Each minicolumn $C_{j}$ has a binary state variable $A_{j}$ and can have potential synapses (connections) to the part of the input space [44] or a sparse set of feed-forward connections from the dimensions of the input [35].

In [44], the Hebbian rule ensures that synaptic permanences are adjusted accordingly. The synapse is only connected if the permanence is greater than the threshold required to connect. To ensure that most of the inputs are active within the local inhibition rule, a local mechanism enables a small faction of minicolumns.

In [35], the authors proposed a simplified method to calculate the activation of the minicolumns as follows:

(6) $$A_{j}^{t}=1\Leftrightarrow \sum_{m\in F_{j}}I_{m}^{t}\geq \theta$$

The SP output represents the activation of minicolumns in response to feedforward inputs.

Meanwhile, the temporal memory learns a sequence and forms a representation in the context of previous inputs. Basically, the algorithm determines the active cells of the columns and learns distal synaptic permanence.

A set of possible synapses is generated at random from a division of cells in the layer within the distal portion. In addition, the permanence of these are also chosen at random.

Equation (7) presents the active state, where we assume that a group $C^{t}$ of columns for the inhibitory process has already been chosen that is most appropriate to compliment the current feed-forward inputs.

It is assumed that an inhibitory process has already selected a set $C^{t}$ of columns that best match the current feed-forward input pattern. The calculation of the active state is given by the following equation:

(7) $$a_{i,j}^{t}=1\Leftrightarrow A_{j}^{t}\wedge \left(\pi_{i,j}^{t-1}\vee \left(\forall m:\neg \pi_{m,j}^{t-1}\right)\right)$$

A cell is activated in a winning column if this cell was predicted ($\pi_{i,j}^{t-1}=1$) or if no cells in this minicolumn are predicted. In the latter case, the minicolumn will experience “bursting”, and thus all cells will be activated. The algorithm will select one of the cells in the learning process.

The predictive state for the current time step is calculated as the following equation:

(8) $$\pi_{i,j}^{t}=1\Leftrightarrow \exists_{k}{∥{\tilde{D}}_{i,j}^{k}◦A^{t}∥}_{1}>\theta$$

where θ represents the spiking threshold and ○ is an element-wise multiplication (Schur product). A cell will be depolarized if at least one segment *k* is active, which occurs if there are more than θ connected synapses with active presynaptic cells.

The learning process uses a Hebbian-like rule. It reinforces the dendritic segment that was responsible for activating and causing the depolarization. The choice of those segments $D_{i,j}^{k}$ is such that

(9) $$\forall A_{j}^{t}\left(\pi_{i,j}^{t-1}>0\right)\wedge {∥{\tilde{D}}_{i,j}^{k}◦A^{t-1}∥}_{1}>\theta$$

It means that the first term selects winning columns that contain correct predictions and the second one selects those segments specifically responsible for the prediction [36].

At this moment, the algorithm will select one of the cells as a learning cell, i.e., the winner cells $w_{i,j}^{t}$, as follows:

(10) $$w_{i,j}^{t}=1\Leftrightarrow A_{j}^{t}\wedge \left(\pi_{i,j}^{t-1}>0\right)\wedge {∥{\tilde{D}}_{i,j}^{k}◦A^{t-1}∥}_{1}>\theta$$

If “bursting” takes place, one of the activated cells is selected on this minicolumn. This cell will represent the context in the future if the current sequence transition repeats:

(11) $$\begin{array}{cc} w_{i,j}^{t}=1\Leftrightarrow & A_{j}^{t}\wedge \left(\forall m:\neg \pi_{m,j}^{t-1}\right)\wedge {∥{\dot{D}}_{i,j}^{k}◦A^{t-1}∥}_{1}=\\ \; & max_{i}\left(∥{\dot{D}}_{i,j}^{k}◦A^{t-1}{∥}_{1}\right) \end{array}$$

where the function $max_{i}$ will select the cell with the segment that was closest to being active, even though it was below the threshold.

The synaptic permanence value is adjusted to reward synapses with active presynaptic cells and punish synapses with inactive cells as follows:

(12) $$\Delta D_{i,j}^{k}=r\left({\dot{D}}_{i,j}^{k}◦A^{t-1}\right)-\gamma {\dot{D}}_{i,j}^{k}$$

where *r* and γ values will increase and decrease all the permanence values corresponding to presynaptic cells.

In [34], the authors eliminated the dendrite segments and replaced the Hebbian-like learning with one-shot learning. This means that k=0, permanence values are always equal to a maximum value, and θ=0. They set the variable $\pi_{i,j}^{t}$ if there is an active cell $a_{m,n}$ with a lateral predictive connection to this cell $c_{i,j}$:

(13) $$\pi_{i,j}^{t}=1\Leftrightarrow {∥{\tilde{D}}_{i,j}◦A^{t}∥}_{1}>0$$

In this case, the set of predictive connections is updated based on the previous and current winner cells:

(14) $$P=P\cup \left\{\left(c_{m,n},c_{i,j}\right):w_{m,n}^{t-1}\wedge w_{i,j}^{t}\wedge \left(∄l:\left(c_{l,n},c_{i,j}\right)\in P\right)\right\}$$

$P\subset \left\{\left(c_{m,n},c_{i,j}\right):i,j,m,n\in N\right\}$

(15) $$w_{i,j}^{t}=1\Leftrightarrow \left(a_{i,j}^{t}\wedge p_{i,j}^{t}\right)\vee b_{i,j}$$

The output of HTM temporal memory represents the activation of individual cells across all minicolumns.

### 2.2. Encoder

Initiating the application of an HTM system involves the initial phase of transforming a data source into a Sparse Distributed Representation (SDR) through a process known as encoding [45]. The encoding process mirrors the functions performed by sensory organs in humans and other animals.

According to Sünderhauf et al. [15], the features generated by Convolutional Neural Networks (CNNs) outperform other methods for the task of visual place recognition in robotics. Despite being trained for a highly specific target task, these models can be effectively applied to deal with different problems. The authors demonstrated that deep features from different layers of CNNs consistently perform better than the traditional system as SIFT or SURF. Additionally, they established important results from which the features are extracted; higher layers of the CNN hierarchy encode semantic information, middle layers exhibit robustness against appearance changes, and top layers are more robust with respect to viewpoint changes.

Indeed, CNNs have become fundamental tools in computer vision, and their architecture is inspired by the visual-processing systems in biological organisms [46]. The success of CNNs has contributed to our understanding of how the brain processes visual information and has enabled the exploration of the connections between artificial and biological intelligence.

Based on [35], the method uses the *conv3* layer of the pretrained CNN AlexNet [47]. This network consists of eight layers: five convolutional layers, two fully connected hidden layers, and one fully connected output layer. The length of the AlexNet-based descriptor is $l_{cnn}=64,896$ and can be excessive for HTM Spatial Pooler performing the SDR transformation. Because of that, we use dimensionality reduction techniques based on the random projection [15] and binarization by using the method proposed in [35], namely binary locality-sensitive hashing (LSH).

### 2.3. Spatial-View-Cells Module

**Figure 6:**
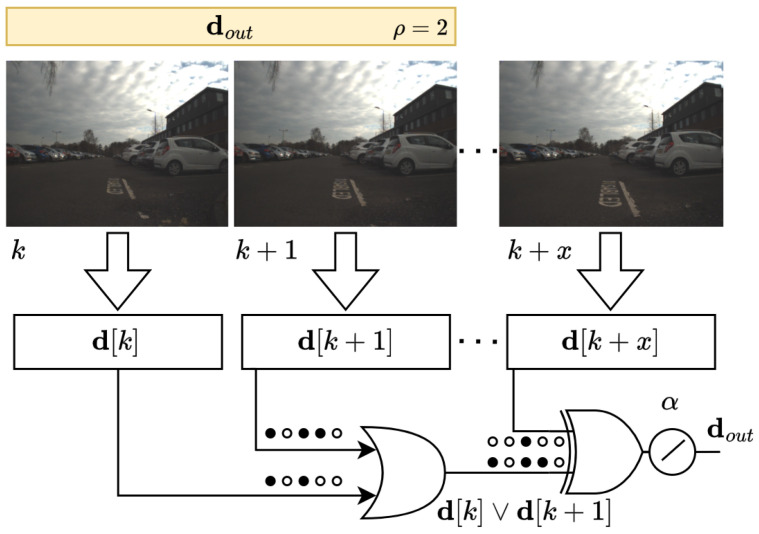
Spatial-view-cell model based on SDR properties and the neurons in primates’ hippocampus that respond when a certain part of the environment is in the animal’s field of view.

This module models spatial-view cells that fire whenever the robot views a certain part of the environment, as primates do. It is responsible for resetting the accumulative errors of the odometry. Each cell represents what the robot is perceiving, similar to the RatSLAM system. However, when a novel visual scene is seen, a new view cell is not necessarily created and associated with the HTM descriptor, as can be seen in Figure 6.

We took advantage of the sparsely distributed representation properties. In [48], Ahmad and Hawkins state that empirical evidence demonstrates that every region of the neocortex represents information using sparse activity patterns at any point in time, and the sparsity might vary from less than one percent to several percent of the total neurons. In other words, SDRs are vectors with thousands of bits and at any point in time a small percentage of the bits are 1’s and the rest are 0’s. The meaning of the features is represented in the set of active bits. As a consequence, if two different descriptors have an active bit in the same location, they share the same attribute.

Therefore, we can take advantage of an important property of SDRs that is related to the union. It is possible to take a set of SDRs and form a new SDR and maintain their attributes of them. For this, we simply make an OR operation, and the result is compared to determine if it is a member of the set of SDRs used to form the union.

Given a set D that represents a sequence of SDR descriptors, defined as

(16) $$D=[d\left[k\right],d\left[k+N_{0}\right]],$$

where $d\in S^{n}$ is defined in Section 2.1.1 and represents the images taken at a specific place and at particular instants in discrete-time k∈N, and $N_{0}$ is an interval defined by

(17) $$N_{0}=\left[0,x\right]=\left\{x\in N:\Phi (k+x,k+x+1)\geq \alpha \right\}$$

The output of this model is the descriptor

(18) $$d_{out}=d\left[k\right]\vee d\left[k+N_{0}\right],$$

The parameter α can be understood as the maximum overlap in order to control the sparsity of the results.

Additionally, we define the parameter ρ as the maximum size of the interval of the spatial-view cells that enclose representations with the similarity between SDRs, i.e.,

(19) $$N_{0}=\left[0,\min(x,\rho )\right]$$

This method helps to avoid the corridor problem, i.e., the absence of a significant structure along both outdoor and indoor environments.

### 2.4. Pose Cell Network

The Pose Cell Network is designed to simulate the functioning of neurons in the rodent brain, specifically those found in the hippocampus and entorhinal cortex. These neurons, known as pose cells, are thought to play a fundamental role in spatial representation and navigation. In the RatSLAM system, the Pose Cell Network emulates the rat’s ability to create a cognitive map of its environment by encoding information about the animal’s position and orientation [49].

A three-dimensional continuous attractor network models the pose cells, where the dimensions represent the pose (i.e., (x,y,θ)) of a ground-based robot with activity matrix *P*.

As the animal explores its environment, these pose cells fire in a manner that creates a unique neural signature for different locations and orientations. The integration of these pose cell activations over time forms a neural representation of the spatial layout, allowing the RatSLAM system to build and update a map of the environment in a self-supervised manner [23].

### 2.5. Experience Map

Pose cells represent a finite area; therefore, a single cell is represented by multiple physical places via the wrapping behavior of the cube’s edges.

An estimate of the robot’s global pose is provided by the experience map and is created by the combination of spatial-view and pose cells.

The map results in a graph where each node represents a different state in the spatial-view and pose cells. When the state of these components changes and is therefore not matching with any pre-existing node in the graph, a new experience node is created within the graph. With these transitions between experiences, links are formed between the current and previous nodes [23].

## 3. Experimental Validation

In this section, we present the experimental procedure for evaluating the NeoSLAM. To evaluate the system performance, we implemented it in two different robots by using the Robot Operating System (ROS Melodic) in three different environments.

The NeoSLAM project deals with complex software tools and dependencies, making it challenging to ensure consistent results across different computing environments (available in https://github.com/cappizzino/neoslam_ws). To address this issue, a Singularity version 3.9.5 container was integrated.

Singularity allows one to create containerized environments that encapsulate all the dependencies and configurations needed for ROS applications. This makes it easy to share ROS-based projects across different systems and ensures consistent behavior regardless of the host environment.

Besides, it is possible to define the exact software versions, libraries, and configurations required for ROS project within the container. This ensures that the code behaves consistently across different development and deployment stages.

Singularity containers can be shared with other researchers and developers, facilitating collaboration in the robotics community. This is particularly useful when working on open-source ROS projects or when collaborating on research initiatives.

Next, we describe the experimental setup and the evaluation metrics used.

### 3.1. Experimental Setup

The first robot used in order to evaluate our model was the Clearpath Husky A200. Husky is an Unmanned Ground Vehicle (UGV), developed by Clearpath Robotics. It is a differential-drive-wheeled robot that has a top speed of 1.0 m/s. The robot is equipped with a bumblebee stereo camera mounted on the pan tilt unit where only the left camera was utilized for collecting images.

The second UGV is a hay-cleaning robot equipped with a simple Microsoft LifeCam VX-700, Rion AH200C IMU, and C16 LS LiDAR, which realizes 360° three-dimensional scanning with 16 laser beams. The robot has differential wheel drive and an onboard computer running Ubuntu. With this robot, we decided to build a map by using the LiDAR-Inertial Odometry Smoothing and Mapping (LIO-SAM) ROS package, a state-of-the-art LiDAR SLAM method, in order to create quite accurate ground truth data.

### 3.2. Environments

In the evaluation of the neocortex model, three different environments were selected to verify and validate the effectiveness of the model created, namely a Robotarium, Heriot-Watt University campus, and a cow barn.

#### 3.2.1. Robotarium

**Figure 7:**
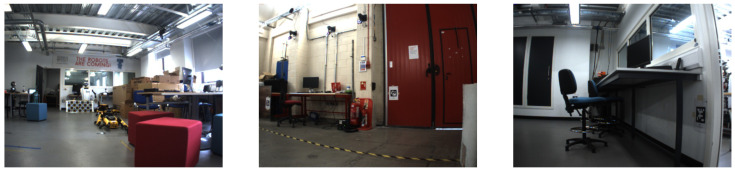
Images collected by the Dual UR5 Clearpath Husky robot at the Heriot-Watt University indoor Robotarium.

Figure 7 shows the Robotarium environment, which is a multipurpose robotics laboratory at the Edinburgh Centre for Robotics (HWU). The area is very cluttered, consisting of several workstations with desks and chairs, robots positioned around the room, etc.

#### 3.2.2. Heriot-Watt University

**Figure 8:**
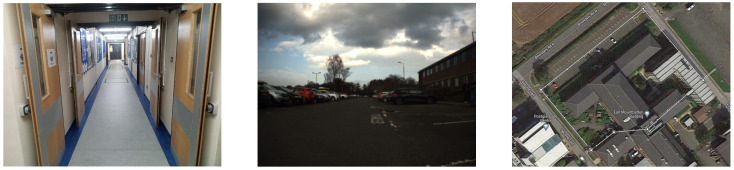
A selection of images of the environment within the Heriot-Watt University campus where the furthest right image displays the route that the robot was teleoperated throughout.

We evaluated our method at Heriot-Watt University, which includes both outdoor and indoor areas. The indoor corridors consisted of a clear area with posters positioned along the walls of the corridor (Figure 8). There were a number of fire doors that were opened ahead of the robot. This area represents a challenge for the neocortex model as many of the images captured are very similar. Meanwhile, the outdoor environment is made up of roads, sidewalk, and car-parking areas.

#### 3.2.3. Cow Barn on the Farmland

**Figure 9:**
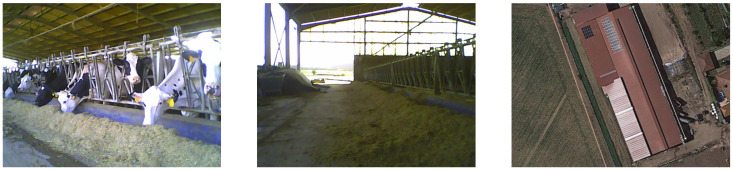
Images and environment from the cow barn on the farmland.

The cow barn is an open-sided structure where cattle eat, as can be seen in Figure 9. The main area of the barn is filled with bales of hay stacked and includes individual stalls for cows and a dedicated feeding area.

### 3.3. Evaluation Metrics

Establishing metrics for visual place recognition typically involves defining evaluation criteria that assess the performance and precision of the recognition system. Some common metrics for VPR include

- Precision (*P*);
- Recall (*R*); and
- Area under the precision–recall curve (AUC).

Precision (*P*) measures the proportion of correctly identified positive instances (true positives—TP) out of the total instances identified as positive, including the false positives (FP). It quantifies the precision of positive predictions made by a VPR system. In other words, precision answers the question: “Of all the items the system identified as positive, how many were actually positive?”:

(20) $$P=\frac{\#TP}{\#TP+\#FP}$$

Recall (*R*), also known as the sensitivity or the true positive rate, measures the proportion of correctly identified positive instances out of all the actual positive instances, including false negatives (FN). It quantifies the system’s ability to find all the relevant positive instances. In other words, recall answers the question: “Of all the actual positive items, how many did the system identify correctly?”:

(21) $$R=\frac{\#TP}{\#TP+\#FN}$$

In order to illustrate the trade-off between precision and recall for a classification model, a precision–recall curve can be generated by plotting these pairs of precision and recall values for various thresholds. In a precision–recall curve, different decision thresholds are used to classify data points, and for each threshold, precision and recall values are calculated.

The area under the precision–recall curve (AUC) is a numerical value that quantifies the overall performance of a classification model as represented by its precision–recall curve. It summarizes the classifier’s performance in a single scalar value, with a higher AUC indicating better performance.

### 3.4. General Procedures

The table below shows the general configuration of the experiments (Table 1).

In Experiment 1 and 2, the images were collected by the Husky robot during the teleoperation. We ensure real-time running, i.e., images were collected while the full data-processing pipeline for loop-closure detection was running. In these cases, odometry data were recorded with 10 Hz and images with approximately 1.0 Hz. In the last experiment, ground truth data were created by using a LiDAR-based SLAM system. Places with a ground truth distance <3 m are considered as the same place.

### 3.5. Parameter Configurations

CNN features were computed by a pretrained AlexNet by using Pytorch. The neocortex model was implemented in Python 2.7.17 by using the Numenta Platform for Intelligent Computing (NuPIC) developed by Numenta, which implements the HTM learning algorithms. We implemented both Spatial Pooler and Temporal Memory algorithms to create temporal and spatial representations of the images. A description of the HTM’s parameters is given in [50] and their values can be found in the project repository.The main parameters’ values can be seen in the table below (Table 2).

## 4. Results

In this section, we discuss the results obtained by using the proposed NeoSLAM method in the delineated experimental scenarios. We validate the following real-world experiments by using evaluation metrics compared to the original RatSLAM.

### 4.1. Robotarium

**Figure 10:**
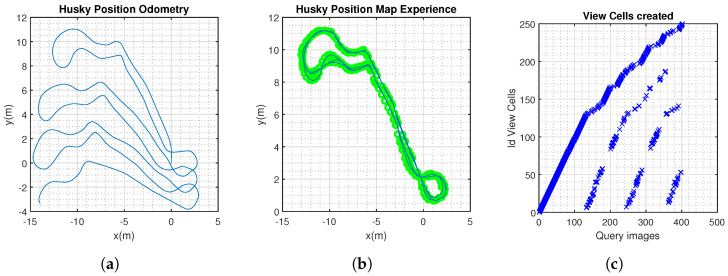
NeoSLAM results. (**a**) Odometry information from Husky robot. (**b**) Experience map, showing the topological map. (**c**) Visual templates over the duration of the experiment.

**Figure 11:**
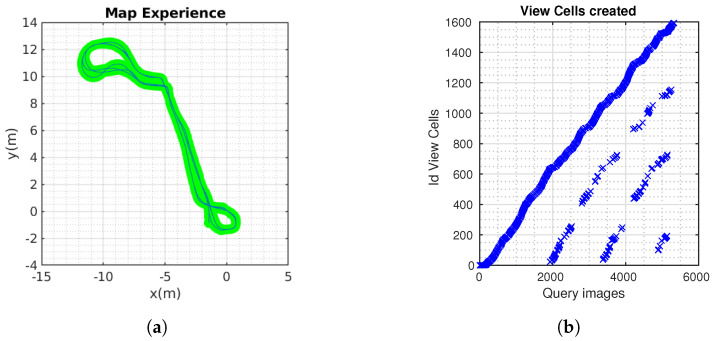
RatSlam results. (**a**) Experience map, showing the topological map. (**b**) Visual templates over the duration of the experiment.

**Figure 12:**
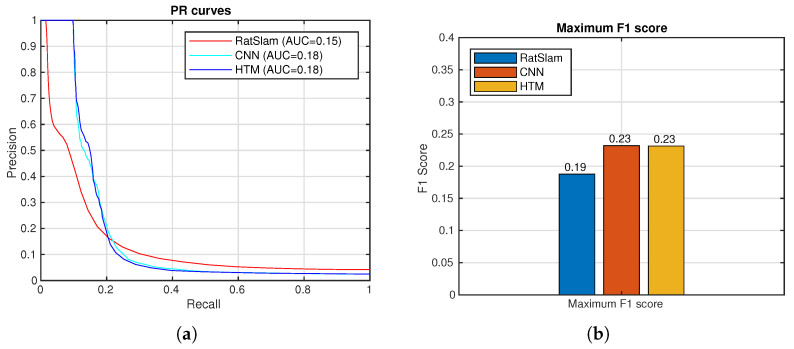
Spatial-view cell NeoSLAM (HTM), CNN, and view-cell RatSLAM descriptors. (**a**) Precision–recall curves. (**b**) Maximum F1 Scores.

**Figure 13:**
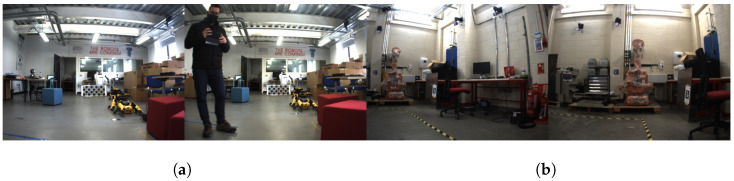
NeoSlam results: examples of true and false positive places. (**a**) True positive images. (**b**) False positive images.

In this scenario, NeoSLAM and RatSLAM generated a consistent topological map of the environment. The algorithms demonstrated real-time performance, processing sensor data and updating the map and trajectory estimates on the fly (Figure 10, Figure 11, Figure 12 and Figure 13).

The SLAM algorithms proved to be robust against sensor noise, occlusions, and dynamic objects. It successfully handled scenarios with moving objects, such as people walking in the environment, without significant degradation in performance.

### 4.2. Heriot-Watt University

**Figure 14:**
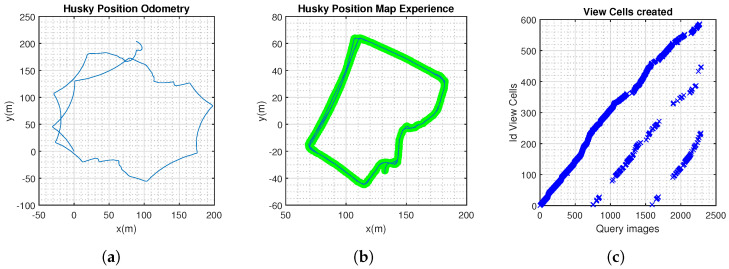
NeoSLAM results. (**a**) Odometry information from Husky robot. (**b**) Experience map, showing the topological map. (**c**) Visual templates over the duration of the experiment.

**Figure 15:**
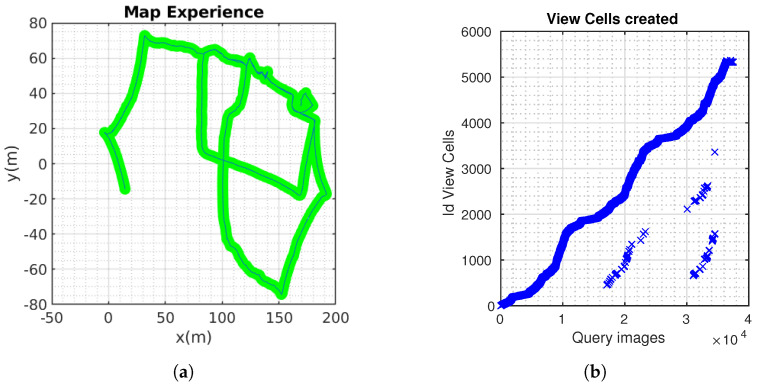
RatSlam results. (**a**) Experience map, showing the topological map. (**b**) Visual templates over the duration of the experiment.

**Figure 16:**
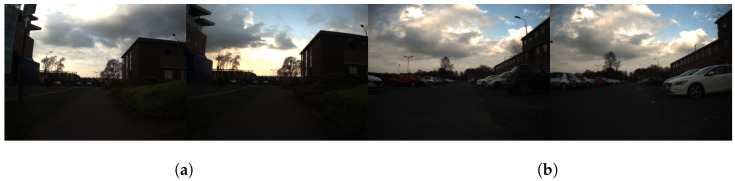
NeoSlam results: examples of true and false positive places. (**a**) True positive images. (**b**) False positive images.

Husky completed three laps, approximately 1.1 km. The route was completed at different times during the day to ensure that the model was tested in different daylight hours. This would assess the resilience of the neocortex model in different lighting: afternoon and evening (Figure 14, Figure 15 and Figure 16).

The transitions between outdoor and indoor environments multiple times, simulating a scenario where a robotic system needs to adapt to changing conditions, aims to assess the adaptability and robustness of our approach across diverse and dynamic scenarios. In total, 2504 images were analyzed. Even so, the system was capable of running in real time without the loss of information due to the possibility of comparing by using the hamming distance.

### 4.3. Cow Barn

In this scenario, cows are moving and eating in different places. The illuminations exhibit significant variation at the middle and extremity of the barn.

**Figure 17:**
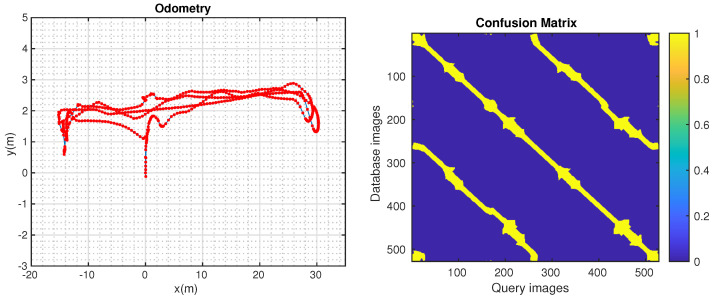
LIO-SAM odometry and confusion matrix.

**Figure 18:**
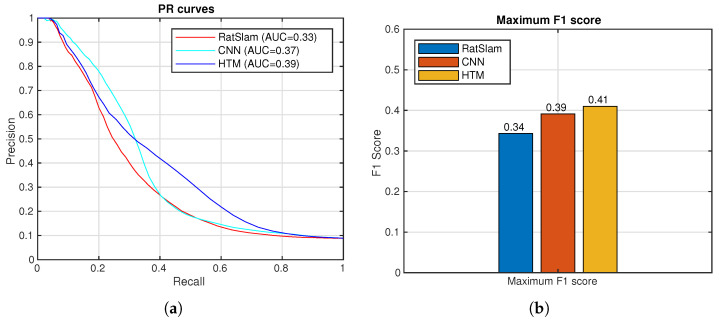
Spatial-view-cell NeoSLAM (HTM), CNN, and view-cell RatSLAM descriptors. (**a**) Precision–recall curves. (**b**) Maximum F1 Scores.

Figure 17 (left) shows the trajectory built by LIO-SAM [51]. The ground truth is calculated and is shown in Figure 17 (right). The robot completed three laps, approximately 400 m. The comparison between the methods is shown below (Figure 18).

## 5. Discussion and Conclusions

The aim of this work was to propose a new visual-based SLAM method using a computational model that integrates neocortex and hippocampus models to deal with environments where appearance continually changes. Our approach focuses on real-world robot implementations, ensuring the relevance and applicability of the findings to practical robotics applications.

Through three distinct experiments, the performance of traditional RatSLAM and NeoSLAM are compared. RatSLAM relies on feature extraction and matching in visual data, which can be affected by lighting conditions, occlusions, and environmental changes, and NeoSLAM is proposed to deal with this problem. The proposed method, with the inclusion of the neocortex model and spatial-view cells for visual place recognition and loop-closure detection, outperforms RatSLAM.

Visual place recognition can benefit immensely from incorporating features learned by using CNNs. Nevertheless, these approaches are computationally intensive, so it is necessary to examine the capability of real-time operation. Using a pretrained CNN is an important alternative from the aspects of reducing the computational cost.

Representations in HTM are binary sparse distributed, operations on these structures are very efficient related to time, and the features obtained from CNNs can be incorporated. Besides, the sequential information that this model incorporates might be a key element of successful approaches for place recognition in changing environments.

In the Robotarium scenario, we did not observe a significant improvement in spite of the fact that the area under the precision–recall curve of the HTM model was slightly higher than RatSLAM. It is important to note the number of view cells created in RatSLAM is approximately ten times higher than the spatial-view cells of NeoSLAM, which completes the analysis of the images every one second.

In the second experiment undertaken at Heriot-Watt University, the results of NeoSLAM overcome the performance of RatSLAM. The complexity of the scenario, the strong similarity of places, and the appearance changes caused a lot of false positives, which hamper the construction of a topological map. These results align with the hypothesis that the incorporation of CNNs into HTM generates distinctive features, contributing to enhanced long-term visual place recognition.

In the final scenario, a ground truth was created by using LIO-SAM, which works by receiving data from a 3D LiDAR and an IMU in order to estimate the robot’s state and trajectory. It formulates the problem as a Maximum a Posteriori Probability (MAP) estimate by using a factor graph to solve it. By doing this, a comparison between NeoSLAM and RatSLAM improves as they can be compared directly for the same types of data in this case. Once again, NeoSLAM obtained a better result.

The evidence presented in this study supports the effectiveness of the proposed NeoSLAM method in achieving consistent topological mapping and a real-time performance in real-world indoor and outdoor environments. NeoSLAM showcases robustness against various challenges, making it suitable for practical robotic applications.

The scope of this study was limited in terms of the changes in the robot’s environment caused by continuously changing appearances. A future study could assess the long-term effects of dark environments and the degree of change in viewpoint, which can significantly impact the challenge associated with VPR. Besides, some limitations were observed during the experiments regarding the complexity and number of parameters of this model. The algorithm has theoretical limitations that require further investigation.

In conclusion, this study not only builds upon the foundational knowledge established by previous works but also offers practical applications. We consider the performance to be an attractive choice for adaptation within field robot applications. These findings contribute to the advancement of SLAM techniques, mainly for long-term robot operation.

## Figures and Tables

**Table 1:** Configuration of the experiments.

Experiment	Robot	Environment	Ground Truth
1	Clearpath Husky	Robotarium	Annotated manually
2	Clearpath Husky	Robotarium	Annotated manually
3	Hay-cleaning robot	Cow barn	LiDAR-based SLAM

**Table 2:** Main parameters for Spatial Pooler, Temporal Memory and Spatial-View Cells module.

Parameter	Module	Robotarium	HWU	Cow Barn
input-, columnDimensions	Spatial Pooler	2048	2048	2048
numActiveColumnsPerInhArea	Spatial Pooler	40	40	40
columnDimensions	Temporal memory	2048	2048	2048
cellsPerColumn	Temporal memory	32	32	32
activationThreshold	Temporal memory	4	4	4
maximum size of the interval ρ	Spatial-view cells	1	3	2
maximum overlap α	Spatial-view cells	-	384	384

## Data Availability

The data presented in this study are openly available in https://github.com/cappizzino/neoslam_ws.

## Abbreviations

The following abbreviations are used in this manuscript:

BC	Border Cells
BoVW	Bag-of-Visual-Words
BRIEF	Binary Robust Independent Elementary Features
BRISK	Binary Robust Invariant Scalable Keypoints
CANN	Continuous Attractor Neural Network
CNN/ConvNet	Convolutional Neural Network
FAB-MAP	Fast-Appearance-Based Mapping technique
FAST	Features from accelerated segment test
GC	Grid cells
HD	Head-direction cells
HP	Hippocampus
HTM	Hierarchical temporal memory
LCD	Loop-closure detection
LIO-SAM	LiDAR-Inertial Odometry Smoothing and Mapping
MAP	Maximum a Posteriori Probability
MEC	Medial Entorhinal Cortex
ORB	Oriented FAST and Rotated BRIEF
ORE	Offshore Renewable Energy sector
PC	Place cell
ROS	Robot Operating System
SLAM	Simultaneous Localization and Mapping
SDR	Sparse distributed representations
SIFT	Scale-Invariant Feature Transform
SP	Spatial Pooler
SURF	Speeded-Up Robust Features
TM	Temporal memory
VLAD	Vector of Locally Aggregated Descriptors
VPR	Visual place recognition
